# The Role of Dopamine in Anticipatory Pursuit Eye Movements: Insights from Genetic Polymorphisms in Healthy Adults

**DOI:** 10.1523/ENEURO.0190-16.2016

**Published:** 2017-01-10

**Authors:** Jutta Billino, Jürgen Hennig, Karl R. Gegenfurtner

**Affiliations:** Department of Psychology, Justus-Liebig-Universität, 35394 Giessen, Germany

**Keywords:** anticipation, COMT, dopamine, genetic polymorphisms, individual differences, pursuit system

## Abstract

There is a long history of eye movement research in patients with psychiatric diseases for which dysfunctions of neurotransmission are considered to be the major pathologic mechanism. However, neuromodulation of oculomotor control is still hardly understood. We aimed to investigate in particular the impact of dopamine on smooth pursuit eye movements. Systematic variability in dopaminergic transmission due to genetic polymorphisms in healthy subjects offers a noninvasive opportunity to determine functional associations. We measured smooth pursuit in 110 healthy subjects genotyped for two well-documented polymorphisms, the COMT Val^158^Met polymorphism and the SLC6A3 3′-UTR-VNTR polymorphism. Pursuit paradigms were chosen to particularly assess the ability of the pursuit system to initiate tracking when target motion onset is blanked, reflecting the impact of extraretinal signals. In contrast, when following a fully visible target sensory, retinal signals are available. Our results highlight the crucial functional role of dopamine for anticipatory, but not for sensory-driven, pursuit processes. We found the COMT Val^158^Met polymorphism specifically associated with anticipatory pursuit parameters, emphasizing the dominant impact of prefrontal dopamine activity on complex oculomotor control. In contrast, modulation of striatal dopamine activity by the SLC6A3 3′-UTR-VNTR polymorphism had no significant functional effect. Though often neglected so far, individual differences in healthy subjects provide a promising approach to uncovering functional mechanisms and can be used as a bridge to understanding deficits in patients.

## Significance Statement

Although the neuronal bases of oculomotor control are well documented, the modulating role of neurotransmitters has remained elusive. Oculomotor deficits have been reported in diseases characterized by disturbed neurotransmission; however, clinical findings lack specificity and are ambiguous because of confounding issues. We used genetic polymorphisms in healthy subjects as an elegant way to investigate the effect of individual differences in dopaminergic circuitry on smooth pursuit eye movements. We found a specific impact of prefrontal dopamine on high-level, but not on low-level, processes involved in pursuit. Our results provide an immediate link between dopamine and oculomotor control in humans. They highlight the value of individual differences for uncovering functional processes and provide insight into the mechanisms underlying oculomotor phenotypes of diseases.

## Introduction

The neural circuits involved in oculomotor control are well described (Krauzlis, 2005), but understanding of the role of neurotransmitters in the fine regulation of sensorimotor processes has remained elusive so far. Dopamine in particular has been discussed as a functionally significant neurotransmitter because of pronounced oculomotor deficits in patients with schizophrenia (Calkins et al., 2008), a disease for which a dysfunction of dopaminergic transmission is considered to be the major pathologic mechanism (Abi-Dargham and Moore, 2003). However, clinical findings are mixed, and links to dopamine are tenuous.

Direct support for the link between dopamine and oculomotor control has recently come from neurophysiology studies in monkeys (Noudoost and Moore, 2011). By pharmacological manipulation of prefrontal dopaminergic transmission, it was possible to modulate visual cortical signals in area V4 that contribute to saccadic target selection. The crucial question that has not been sufficiently answered yet is whether dopaminergic modulation of eye movements can also be seen in healthy human observers. Pharmacological challenge studies indicate that application of dopamine antagonists can disrupt smooth pursuit eye movements (Malaspina et al., 1994; Reilly et al., 2008), but specificity of deficits and reliability of results remained unclear. An alternative noninvasive way to investigate behavioral effects of dopamine in humans is provided by genetic polymorphisms functionally associated with neurotransmission.

**Figure 1.**
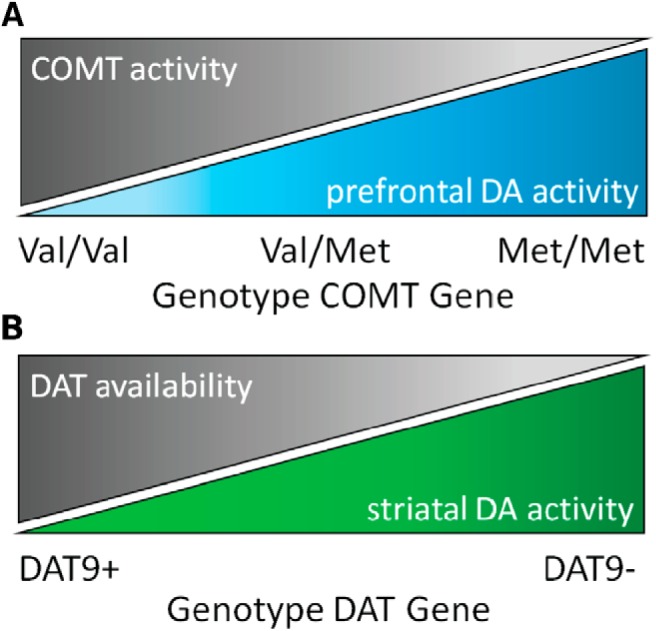
Putative functional mechanisms of dopaminergic polymorphisms. ***A***, COMT Val^158^Met polymorphism: genotype modulates activity of the COMT enzyme that represents the major dopamine breakdown mechanism in prefrontal cortex; lower enzyme activity results in higher prefrontal dopamine activity. ***B***, SLC6A3 3′-UTR-VNTR polymorphism: genotype modulates availability of the DAT that terminates striatal dopamine activity by fast reuptake from the synaptic cleft; lower availability of the transporter results in higher striatal dopamine activity.

The COMT (catechol-*O*-methyltransferase) Val^158^Met polymorphism [rs4680] and the SLC6A3 3′-UTR-VNTR polymorphism [rs28363170] represent two well-studied dopaminergic polymorphisms (Vandenbergh et al., 1992; Lachman et al., 1996). Fig. 1 illustrates their proposed functional mechanisms.

Activity of the dopamine-degrading enzyme COMT is modulated by a single-nucleotide polymorphism. The encoding gene is subject to a mutation that results in a substitution of methionine (Met) for valine (Val) at codon 158. The Met allele is associated with reduced enzyme activity, leading to less efficient dopamine catabolism. In Val/Val homozygotes, a three- to four-fold higher activity than in Met/Met homozygotes is observed; Val/Met heterozygotes show intermediate activity levels (Lachman et al., 1996). Less active COMT contributes to higher dopamine levels. This mechanism is dominant in prefrontal cortex because of a local lack of alternative breakdown mechanisms (Tunbridge et al., 2006). In striatal regions, dopamine regulation relies on the dopamine active transporter (DAT) that terminates dopamine activity by reuptake from the synaptic cleft. Its availability is modulated by a variable number of tandem repeats polymorphism of the 3′-untranslated region (UTR) of the encoding gene (Vandenbergh et al., 1992; Costa et al., 2011; Faraone et al., 2014). Repeats range between 3 and 13, with 9- and 10-repeat alleles having the highest frequency in the population (Kang et al., 1999). Although the functional mechanism holds some tentativeness, converging evidence from meta-analyses supports higher DAT availability in carriers of the 9-repeat allele. Lower DAT availability in noncarriers putatively slows down reuptake and thereby increases dopamine activity (Costa et al., 2011; Faraone et al., 2014).

Although a variety of behavioral correlates of both polymorphisms have been studied, insights into their role in oculomotor control are sparse. Some findings point to functional associations with antisaccade performance (Ettinger et al., 2008; Haraldsson et al., 2010; Kasparbauer et al., 2015), but differences foremost showed up in BOLD responses. Smooth pursuit seems ideal for studying different mechanisms involved in oculomotor control, because it relies on a sophisticated interplay between motion perception, sensorimotor transformation, and anticipatory abilities (Lisberger, 2010; Kowler et al., 2014). However, the link between both polymorphisms and individual variations in pursuit has not been investigated appropriately yet. Studies focused primarily on pursuit deficits in schizophrenic patients (Rybakowski et al., 2002; Thaker et al., 2004; Haraldsson et al., 2009; Wonodi et al., 2009). Although healthy subjects were included as control groups, procedures might have been not well suited to detect individual differences in the normal range and particularly in different pursuit parameters. Conclusions have been complicated by inconsistent results and insufficient differentiation between specific pursuit mechanisms.

We aimed to shed light on the role of dopamine in oculomotor control by studying the link between dopaminergic polymorphisms and smooth pursuit, specifically in healthy subjects. We acknowledge the complexity of regulatory processes during pursuit by considering selective oculomotor measures that emphasize either the role of low-level or high-level signals involved. This distinction alludes to the specific functional contributions of retinal sensory signals and extraretinal signals, including, e.g., attention, learning, prediction, or anticipation (Yasui and Young, 1975; Robinson et al., 1986; compare also Kowler et al., 2014). Low-level signals drive bottom-up control, whereas high-level signals account for top-down control. We investigated their associations with two polymorphisms that tap different dynamics of dopaminergic transmission.

## Materials and Methods

### Participants

A total of 110 subjects (18 males) participated in our study. Age ranged from 18 to 45 years, with a mean age of 23.7 years (SD 5.1). Subjects were undergraduate students enrolled in the psychology program at the Justus Liebig University Giessen and fulfilled requirements of the study program with their participation. All students were naive with respect to the purpose of the study. Any history of neurological or psychiatric disorders as well as medications or drug use presumed to interfere with oculomotor functioning were screened out by a comprehensive interview protocol. Methods and procedures agreed with the Declaration of Helsinki (World Medical Association, 2013). Informed consent was obtained by all participants, and protection of data privacy was provided.

### Genotyping

Genetic analyses were conducted within the Gene Brain Behaviour Project (GGBBP) run by the Department of Psychology at the Justus Liebig University Giessen. The project maintains a large subject database characterized by selected polymorphisms functional for neurotransmission and available for behavioral research. All genetic analyses are performed and documented by an experienced technician in the local GGBBP laboratory. DNA was extracted from buccal cells and purified with a commercial extraction kit (MagNAPure LC DNA, Roche Diagnostics).

For the COMT Val^158^Met polymorphism [rs4680], genotyping was accomplished by PCR and fluorescence melting-curve detection analysis. We used the Light Cycler System (Roche Diagnostics, RRID: SCR_001326). Amplification and detection were performed using Light Cycler FastStart DNA Master Hybridization Probes (Roche Diagnostics, RRID: SCR_001326) with the following contents: reaction buffer, dNTPs mix, and Taq DNA polymerase (0.7×) plus 1.6 mm magnesium chloride, 0.6 μm of each of the primers, 0.2 μm of each of the hybridization probes, and ∼50 ng of genomic template DNA. All reactions were carried out in a total volume of 21.4 μl. Primers and hybridization probes (TIB Molbiol) were as follows: forward primer, 5′-GGGCCTACTGTGGCTACTCA-3′; reverse primer, 5′-GGCCCTTTTTCCAGGTCTG-3′; anchor hybridization probe, 5′-LCRed640-TGTGCATGCCTGACCCGTTGTCA-phosphate-3′; sensor hybridization probe, 5′-ATTTCGCTGGCATGAAGGACAAG-fluorescein-3′. The PCR run comprised 53 cycles of denaturation (95°C, 0 s, ramp rate 20°C/s), annealing (57°C, 10 s, ramp rate 20°C/s), and extension (72°C, 10 s, ramp rate 20°C/s), which followed an incubation period of 10 min at 95°C to activate the FastStart Taq DNA polymerase of the reaction mix. The fluorescence signal was plotted against temperature to yield the respective melting points (Tm) of the two alleles. Tm for the Val allele was 59°C, and for the Met allele, 64.5°C.

For the SCL6A3 3′-UTR-VNTR polymorphism [rs28363170], genotyping was accomplished by PCR and gel electrophoresis. DNA amplification reactions were performed as described below, using a Mastercycler EP (Eppendorf, RRID: SCR_000786). PCR amplifications were performed in 25-µl reaction volumes containing ∼50 ng of genomic template DNA, 0.08 U/µl of Qiagen TopTaq DNA polymerase, 1× PCR buffer, and 0.4 mm dNTP mix (Qiagen), 1× BSA, 2× DMSO, and 0.6 µm each of forward (5′-TGTGGTGTAGGGAACGGCCTGAG-3′) and reverse (5′-CTTCCTGGAGGTCACGGC TCAAGG-3′) primers (TIB Molbiol). Thermal cycling consisted of a 7-min initial denaturation phase at 95°C followed by 35 cycles of 95°C (45 s), 63°C (45 s), and 72.8°C (60 s), with a final extension step of 5 min at 72.8°C. Finally, 12 µl of the PCR product was separated by means of gel electrophoresis on a 3% agarose gel in Tris/borate/EDTA buffer (160 V, 60 min) and visualized by ethidium bromide.

Distributions of genotypes for both polymorphisms are given in Table 1. For the SCL6A3 3′-UTR-VNTR polymorphism, we found four subjects who carried an 11-repeat allele. Because these genotypes are extremely rare, such subjects are commonly ignored in association studies. We congruently excluded the 11-repeat allele carriers from further analyses so that our sample was reduced to 106 subjects. Hardy–Weinberg equilibrium tests yielded significant deviations for neither polymorphism.

**Table 1.** Distributions of genotypes and Hardy–Weinberg equilibrium test statistics

Polymorphism	Frequency (*n*) of genotype			*χ* ^2^	*p*
COMT Val^158^Met [rs4680]	Val/Val: 24	Val/Met: 54	Met/Met: 32	0.019	0.89
SCL6A3 3′-UTR-VNTR [rs28363170]	9/9: 8	9/10: 42	10/10: 56	0.001	0.97

Val/Val, homozygote valine/valine; Val/Met, heterozygote valine/methionine; Met/Met, homozygote methionine/methionine; 9/9, homozygote 9-repeat/9-repeat; heterozygote 9-repeat/10-repeat; 10/10, homozygote 10-repeat/10-repeat.

### Pursuit tasks

**Figure 2.**
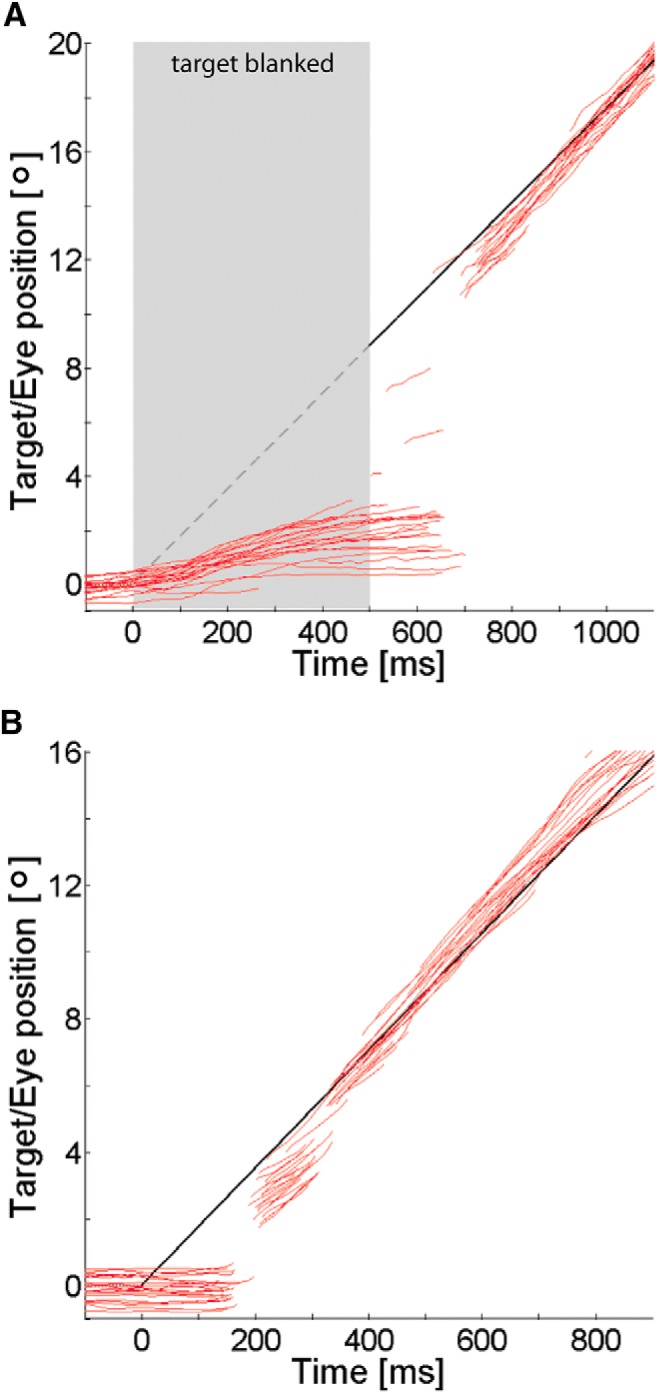
Pursuit paradigms. ***A***, Anticipatory pursuit task: we established a stable expectation of target motion, i.e., fixed direction and velocity; each trial began with a period in which the target trajectory was blanked; the target became visible only after 500 ms. ***B***, Visually guided pursuit task: in a ramp paradigm, the target was visible throughout each trial; motion direction randomly varied from trial to trial; thus motion trajectories were not predictable. Targets moved at a constant velocity of 17.6°/s, and positions are illustrated by black solid and gray dashed lines, respectively; individual eye position traces of an exemplary subject are shown in red.

Smooth pursuit eye movements were recorded in an anticipatory and a visually guided task. In visually guided pursuit, initiation is driven by low-level, retinal signals. During the following steady-state phase, high-level extraretinal signals contribute to pursuit maintenance, but retinal signals are continuously integrated (Yasui and Young, 1975; Robinson et al., 1986). In contrast, anticipatory pursuit is suited to measure specifically high-level mechanisms of oculomotor control because retinal motion signals are eliminated. Fig. 2 illustrates both paradigms by individual eye position traces of an exemplary subject. The pursuit target was a black dot with a diameter of 0.5° moving on a uniform gray background. The luminances for gray and black pixels were 41.4 and 0.18 cd/m^2^, respectively, resulting in a Michelson contrast of 99% for the target.

Our anticipatory task corresponded to the established occluded onset pursuit paradigm (Collins and Barnes, 2006; Freyberg and Ilg, 2008). The target moved at a constant velocity of 17.6°/s horizontally over a distance of 32°. Trials started with an initial fixation period of 500 ms. A fixation dot equivalent to the pursuit target was provided at 16° either left or right from the center of the screen, both randomized and balanced across trials. Fixation dot position indicated direction of subsequent target motion. It started to the right or to the left, respectively, immediately after the fixation period. In a learning phase consisting of 10 trials, subjects were trained to pursue the moving target and were given the opportunity to build up a stable movement expectation. Subsequently, in 50% of a total of 40 trials, the target was blanked for 500 ms after motion onset. The offset of the fixation target here provided the signal for pursuit initiation. Trials with visible target and blanked target were randomly interleaved to stabilize anticipation. To measure visually guided pursuit in a paradigm as similar as possible to the anticipatory paradigm, we used ramp target movements in our visually guided task. Fixation at trial start was set to the center of the screen and varied in duration uniformly between 600 and 1100 ms. This jitter was introduced to avoid anticipatory responses. Next the target started to move horizontally from the center of the screen either to the right or to the left. Target direction was randomized and balanced. Target velocity was held constant at 17.6°/s. Subjects self-initiated trials in both pursuit tasks by pressing the space bar.

### Eye tracking equipment

Stimuli were generated using Matlab (RRID: SCR_001622) with the Psychophysics Toolbox extension (Brainard, 1997, RRID: SCR_002881). They were displayed on a 21-inch SONY GDM-F520 CRT monitor driven by an Nvidia Quadro NVS 290 graphics board with a refresh rate of 100 Hz noninterlaced. The spatial resolution was set to 1280 × 1024 pixels. Subjects were seated in a darkened room at a distance of 47 cm in front of the monitor. Eye movements were registered by a SR Research Eyelink 1000 Tower Mount system (SR Research, RRID: SCR_009602) at a sampling rate of 2000 Hz. Viewing was binocular, and the subject’s head was stabilized by a chin- and headrest. A 9-point calibration was applied, and accuracy was accepted if the procedure yielded values of average error not larger than 0.4° and worst error not larger than 0.7°.

### Data analysis

Genotyping and eye tracking experiments were carried out in parallel and were performed independently. Genetic and behavioral data were combined only after completion of data collection; thus measurement of pursuit was blind to subject genotype.

The COMT Val^158^Met polymorphism was entered in the analyses with three levels according to the genotypes, namely Val/Val, Val/Met, and Met/Met. For the SLC6A3 3′-UTR-VNTR polymorphism, two subgroups were considered. Given the low prevalence of 9-repeat homozygous individuals, subjects were grouped into 9-repeat allele carriers (DAT9+) and noncarriers (DAT9–). Table 2 shows frequencies of the defined levels for both polymorphisms. As mentioned above, four subjects who carried an 11-repeat allele of the SLC6A3 3′-UTR-VNTR polymorphism were excluded from analyses due to the rareness of their genotype.

**Table 2.** Frequency of defined levels for the two polymorphism

		SLC6A3 3′-UTR-VNTR	*n*
COMT Val^158^Met	Val/Val	DAT9+	11
		DAT9–	13
	Val/Met	DAT9+	24
		DAT9–	29
	Met/Met	DAT9+	15
		DAT9–	14
Total		DAT9+	50
		DAT9–	56

Val/Val, homozygote valine/valine; Val/Met, heterozygote valine/methionine; Met/Met, homozygote methionine/methionine; DAT9+, carriers of the 9-repeat allele, DAT9–, noncarriers of the 9-repeat allele.

Eye position traces were analyzed offline. We obtained eye velocity traces by differentiation of position signals over time. Position and velocity traces were smoothed by a Butterworth filter with cutoff frequencies of 30 and 20 Hz, respectively. Saccades were detected using the standard algorithm provided by Eyelink software, which is based on a saccade velocity threshold of 30°/s and a saccade acceleration threshold of 9500°/s. We removed saccades as well as a period of 15 ms before and after saccade onset and offset from the traces. Velocity signals during saccades were replaced by linear interpolation between the velocity before and after each saccade.

As parameters of interest in the visually guided pursuit task, we analyzed pursuit latency, steady-state velocity gain, and pursuit ratio within a time window of 400–700 ms after target motion onset. Pursuit onset was determined for each individual velocity trace by an established procedure using the best fitting regression line in a specified time interval (Schütz et al., 2007). Pursuit ratio has been introduced by Liston and Stone (2014) as a critical steady-state tracking metric and gives the proportion of eye displacement during pursuit relative to the total eye displacement during a given interval. Anticipatory pursuit response was described by maximum eye velocity during the blank period, by average eye velocity in the last 100 ms of the blank period, and by eye position at the end of the blank period. In addition, we analyzed pursuit latency after visible target onset, amplitude of the first catch-up saccade, and distance between its endpoint and target position. The latter metric was termed amplitude lag and provides a measure how far the eye lags behind the target after the first catch-up saccade. Finally, we also considered steady-state velocity gain and pursuit ratio when the target had become visible after the blank period and thereby obtained additional measures for visually guided pursuit.

## Results

We analyzed the association between smooth pursuit parameters and dopaminergic polymorphisms by multivariate ANOVA (MANOVA), with pursuit parameters as dependent variables and levels of the COMT Val^158^Met polymorphism and the SLC6A3 3′-UTR-VNTR polymorphism as independent variables. The COMT Val^158^Met polymorphism was entered with three levels, Val/Val, Val/Met, and Met/Met; the SLC6A3 3′-UTR-VNTR polymorphism was entered with two levels, DAT9+ and DAT9–. If appropriate, follow-up univariate ANOVAs were run separately for each dependent variable. In addition, we explored how well specific parameters allowed classification of genotype groups by receiver operating characteristic (ROC) analyses.

**Figure 3.**
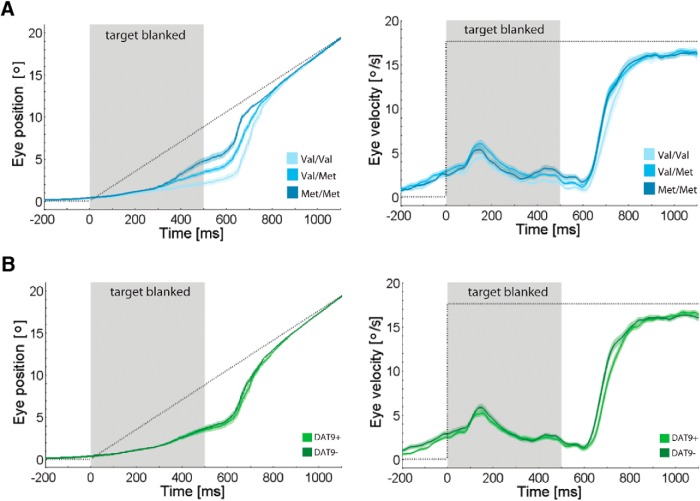
Association between dopaminergic polymorphisms and anticipatory smooth pursuit eye movements. ***A***, Horizontal eye position and velocity traces for different genotypes of the COMT Val^158^Met polymorphism. ***B***, Horizontal eye position and velocity traces for different genotypes of the SCL6A3 3′-UTR-VNTR polymorphism. Note that saccades are removed from our data so that position and velocity traces are not directly comparable. Shaded areas, SEM; dotted lines, target position.

Our data showed that only anticipatory pursuit processes are subject to pronounced dopaminergic modulation, in particular by the COMT Val^158^Met polymorphism. Fig. 3 illustrates eye position and velocity traces in the anticipatory pursuit task for the different genotypes of the COMT Val^158^Met polymorphism and the SLC6A3 3′-UTR-VNTR polymorphism, respectively.

To investigate the effect of the polymorphisms on anticipatory pursuit, we chose the pursuit metrics that are most indicative for the anticipatory response and entered them as dependent variable into a MANOVA. In particular, we considered anticipatory velocity and position during the blank period, but also pursuit latency after visible target onset, amplitude of the first catch-up saccade, and distance between its endpoint and target position. Although the last three metrics apply to an interval in which the target was visible and thus pursuit was also driven by retinal signals, they crucially depend on the purely anticipatory extraretinal response during the period when the target was blanked. Results showed a significant main effect of the COMT genotype (*V* = 0.37, *F*(12,192) = 3.68, *p* < 0.001, η^2^ = 0.19) that explains overall 19% of variance. We found neither a main effect of the DAT factor (*V* = 0.04, *F*(6,95) = 0.67, *p* = 0.677, η^2^ = 0.04) nor an interaction effect of both polymorphisms (*V* = 0.08, *F*(12,192) = 0.63, *p* = 0.817, η^2^ = 0.04).

**Figure 4.**
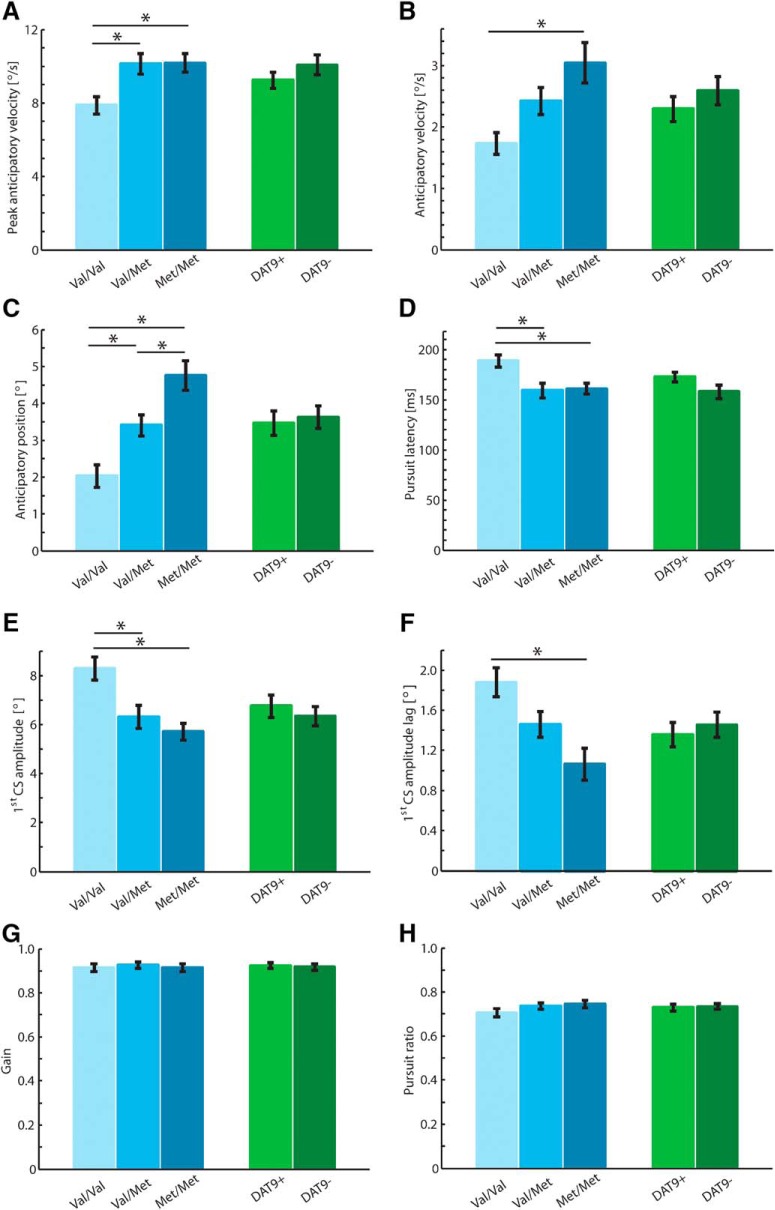
Association between dopaminergic polymorphisms and anticipatory smooth pursuit eye movements. Pursuit metrics are given for the COMT Val^158^Met polymorphism and the SCL6A3 3′-UTR-VNTR polymorphism side by side. ***A***, Peak horizontal eye velocity during the 500-ms blank period when the target was not visible; note that values here differ from the average velocity traces because of temporal variability in individual observers. ***B***, Average horizontal eye velocity in the last 100 ms of the blank period before the target became visible. ***C***, Horizontal eye position at the end of the blank period when the target became visible. ***D***, Pursuit latency after visible target onset. ***E***, Amplitude of first catch-up saccade (CS) after visible target onset. ***F***, Amplitude lag of first catch-up saccade (CS) after visible target onset. ***G***, Velocity gain during steady-state pursuit. ***H***, Pursuit ratio during steady-state pursuit. Error bars: SEM.

Follow-up univariate ANOVAs on separate parameters revealed that individuals with COMT genotypes putatively associated with higher prefrontal dopamine levels, i.e., carriers of the Met allele, showed a boost in anticipatory pursuit response in absence of a visible target (Fig. 4*A–C*). During the anticipatory phase, their eyes initially reached a higher peak velocity, close to 60% of the anticipated target velocity, compared with 45% for Val allele homozygotes (*F*(2,100) = 4.04, *p* = 0.021, *η* ^2^ = 0.08). These peak velocity values are considerably higher than those given by the average velocity traces shown in Fig. 3*A* because the point in time when eye velocity peaked during the blank period substantially varied between individual subjects. However, distribution pattern did not depend on genotype. The advantage was maintained up to visible target onset, when we still found a significant genotype effect on eye velocity (*F*(2,100) = 4.93, *p* = 0.009, η^2^ = 0.09) as well as on eye position (*F*(2,100) = 14.07, *p* < 0.001, η^2^ = 0.22). Met allele homozygotes showed an eye velocity almost twice as high as Val allele homozygotes, i.e., 3.1°/s versus 1.7°/s. In addition, their eye position was closer to the target, at 4.8° versus 2.0°.

After target appearance, the anticipatory advantage of Met allele carriers was carried forward (Fig. 4*D–F*). Pursuit initiation took ∼30 ms less (*F*(2,100) = 4.72, *p* = 0.011, η^2^ = 0.09). Their first catch-up saccade was of smaller amplitude, 6° versus 8° in Val allele homozygotes (*F*(2,100) = 6.17, *p* = 0.003, η^2^ = 0.11). At the same time, it landed closer to the target, resulting in a lag of 1° versus 2° in Val allele homozygotes (*F*(2,100) = 6.59, *p* = 0.002, η^2^ = 0.12). Notably, the advantage was completely cancelled in the steady-state pursuit phase. Neither velocity gain nor pursuit ratio differed between the three COMT genotype groups (Fig. 4*G*, *H*).

As revealed by the initial MANOVA, the SCL6A3 3′-UTR-VNTR polymorphism was not associated with anticipatory pursuit processes. Figs. 3 and 4 illustrate that the anticipatory pursuit response of carriers and noncarriers of the 9-repeat allele completely overlapped. Genotype groups did not differ in any of the anticipatory pursuit metrics.

Given these results, we furthermore performed ROC analyses to investigate how well pursuit parameters differentiate between genotypes of the COMT Val^158^Met polymorphism. Results are summarized in Table 3. The area under the curve (AUC) illustrates classification performance for each metric; a value of 1 corresponds to perfect classification, whereas a value of 0.5 corresponds to random classification. Scores show that anticipatory pursuit parameters consistently allow successful discrimination between Val/Val and Met/Met genotypes as well as between Val/Val and Val/Met genotypes. Val/Met and Met/Met genotype groups, in contrast, showed very similar pursuit patterns, and ROC scores indicate that most parameters discriminate only insufficiently between the two genotypes.

**Table 3.** ROC scores of pursuit metrics predicting COMT genotype by pairs

	Val/Met vs. Met/Met		Val/Val vs. Val/Met		Val/Val vs. Met/Met	
	AUC	95% CI	AUC	95% CI	AUC	95% CI
Anticipatory pursuit task						
Peak velocity	0.56	0.42–0.69	0.70	0.55–0.81	0.73	0.57–0.84
Anticipatory velocity	0.62	0.48–0.74	0.66	0.52–0.78	0.76	0.60–0.86
Anticipatory position	0.70	0.57–0.81	0.72	0.58–0.83	0.86	0.73–0.93
Pursuit latency	0.53	0.40–0.65	0.71	0.57–0.82	0.75	0.60–0.86
First CS amplitude	0.64	0.51–0.75	0.68	0.57–0.79	0.81	0.66–0.90
First CS amplitude lag	0.63	0.49–0.74	0.65	0.51–0.76	0.79	0.64–0.88
Visually pursuit task						
Pursuit latency	0.50	0.36–0.63	0.58	0.43–0.72	0.57	0.40–0.72
Velocity gain	0.54	0.41–0.66	0.58	0.42–0.68	0.64	0.47–0.77
Pursuit ratio	0.54	0.41–0.66	0.60	0.47–0.72	0.66	0.50–0.79

95% CI, 95% confidence intervals for the AUC, estimated using a logit model; CS, catch-up saccade.

**Figure 5.**
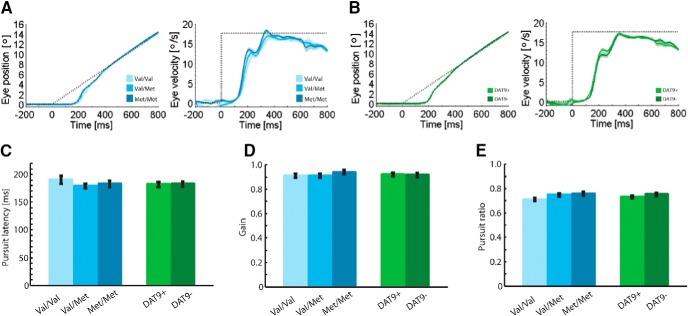
Association between dopaminergic polymorphisms and visually guided smooth pursuit eye movements. ***A***, Horizontal eye position and velocity traces for different genotypes of the COMT Val^158^Met polymorphism. ***B***, Horizontal eye position and velocity traces for different genotypes of the SCL6A3 3′-UTR-VNTR polymorphism. Note that saccades are removed from our data, so position and velocity traces are not directly comparable. Shaded areas, SEM; dotted lines, target position. Pursuit metrics (***C–E***) are given for the COMT Val^158^Met polymorphism and the SCL6A3 3′-UTR-VNTR polymorphism side by side. ***C***, Pursuit latency after target motion onset. ***D***, Velocity gain during steady-state pursuit. ***E***, Pursuit ratio during steady-state pursuit. Error bars: SEM.

Although anticipatory pursuit was clearly modulated by the COMT Val^158^Met polymorphism, visually guided pursuit was not affected by the investigated dopaminergic polymorphisms. Results of the MANOVA with the three parameters of interest in the visually guided pursuit task as dependent variables yielded no significant main effects of the COMT factor (*V* = 0.06, *F*(6,198) = 0.96, *p* = 0.457, η^2^ = 0.03) or the DAT factor (*V* = 0.01, *F*(3,98) = 0.36, *p* = 0.786, η^2^ = 0.01). Also, the interaction of the two polymorphisms did not reach significance (*V* = 0.04, *F*(6,198) = 0.64, *p* = 0.697, η^2^ = 0.02). Fig. 5 demonstrates equivalent visually guided pursuit processes across the defined levels of the COMT Val^158^Met polymorphism and the SCL6A3 3′-UTR-VNTR polymorphism. A direct comparison between classification performance of anticipatory and visually guided metrics for COMT genotypes is provided in Table 3. ROC scores corroborate that only anticipatory pursuit parameters discriminate between COMT genotypes; visually guided pursuit parameters do not provide sufficient classification information.

## Discussion

Directed eye movements represent one of the most fundamental principles of visual information processing in humans. The ability to analyze visual information in detail relies on eye movements that bring selected targets to the fovea and stabilize them there. Although there are comprehensive models of the functionally involved neural pathways (Krauzlis, 2005), the impact of specific neurotransmitters on oculomotor control is still hardly understood. This in particular appears surprising, because there is a long history of eye movement research in patients with psychiatric diseases that are etiologically characterized by a disturbed balance of neurotransmitter activity (Klein and Ettinger, 2008). We studied smooth pursuit eye movements in a large sample of healthy subjects genotyped for well-documented polymorphisms that modulate prefrontal and striatal dopaminergic transmission. We aimed to explore how individual differences in dopamine activity modulate oculomotor control. Different paradigms were used that tapped on low-level and high-level mechanisms underlying smooth pursuit.

Our results highlight the critical functional role of dopamine for anticipatory pursuit, but not for sensory-driven pursuit processes. Whereas our data provide clear evidence that anticipatory pursuit is modulated by dopamine, visually guided pursuit was not affected by dopaminergic polymorphisms. This shows that dopamine is particularly functional for top-down processes involved in pursuit. Visually guided pursuit crucially involves bottom-up processes, so dopaminergic effects are most likely swamped by low-level sensory noise for estimating visual motion (Osborne et al., 2005).

Control of smooth pursuit eye movements is based on a dynamic interplay between low-level retinal and high-level extraretinal signals (Barnes, 2008; Lisberger, 2010; Kowler et al., 2014). Sensory and cognitive contributions are closely interwoven, but rely on differential neuronal resources. We considered the initiation of anticipatory pursuit in particular as a prominent indicator of high-level processes. It has been shown that if upcoming target motion is predictable, anticipatory pursuit can be elicited without visual target information (Hemptinne et al., 2006; Freyberg and Ilg, 2008). The anticipatory pursuit responses we observed in our study were comparable to previous reports, but we found them modulated by individual differences in dopaminergic transmission. In contrast, visually driven pursuit did not systematically vary across individuals. Previous studies that strived to investigate the role of dopamine in different pursuit processes used continuous waveform motion, providing perfectly predictable target trajectories, and blanked the target for a short period during ongoing pursuit (Thaker et al., 2004; Wonodi et al., 2009). Although this approach has yielded seminal insights into pursuit deficits in schizophrenia patients, we suggest that using two separate paradigms for visually guided and anticipatory pursuit allows a more detailed differentiation between underlying mechanisms. In a continuous waveform motion paradigm, pursuit during a blank period clearly relies on extraretinal mechanisms, but when the target is visible, the degree to which visual error signals or predictive signals drive pursuit remains ambiguous. In our paradigms, low-level sensory contributions and high-level anticipation are fully segregated.

In the present study, we considered two functional polymorphisms that modulate the two major factors in regulation of dopamine activity, namely COMT by dopamine catabolism in prefrontal cortex and DAT by dopamine reuptake in striatal regions. For both polymorphisms, COMT Val^158^Met and SCL6A3 3′-UTR-VNTR, elaborate functional models have been derived (Vandenbergh et al., 1992; Lachman et al., 1996), but reliability of behavioral associations has been often discussed controversially.

The COMT Val^158^Met polymorphism has been convincingly associated with prefrontal cortex function (Yavich et al., 2007). Systematic differences between genotype groups were reported for behavioral performance in a variety of cognitive tasks demanding executive control (Tunbridge et al., 2006; Barnett et al., 2008; Goldman et al., 2009) as well as for task-related BOLD activation in prefrontal brain regions (Mier et al., 2010). Furthermore, the COMT polymorphism has been assumed to be associated with various psychiatric diseases, in particular schizophrenia (Lachman et al., 1996). However, initial reports of a significant association between the COMT gene and schizophrenia were not confirmed by later meta-analyses (Egan et al., 2001; Munafo et al., 2005; Okochi et al., 2009). Similarly, a putative link between the COMT polymorphism and specific personality traits, e.g. schizotypy, has to be considered as elusive (Montag et al., 2012; Mohr and Ettinger, 2014). Most likely, the complexity of psychiatric etiologies or personality traits does not allow to determine the specific role of the COMT polymorphism, which might be minor. Our results show that not only performance in typical cognitive tasks, but also sensorimotor control in smooth pursuit is crucially modulated by the COMT genotype. If task characteristics emphasize top-down control in pursuit, substantial effects of genotype are observed. Notably, genotype explained about 19% of variance in the anticipatory pursuit parameters, which points to a much stronger association than typically found for performance in cognitive tasks (compare Egan et al., 2001 and Barnett et al., 2008). Our results also agree with previous preliminary findings on the link between the COMT Val^158^Met polymorphism and pursuit eye movements that have been reported for healthy control subjects in a clinical study in schizophrenia patients. Thaker et al. (2004) showed an advantage in predictive pursuit for healthy Met allele homozygotes and found 10% of variance in the gain parameter to be explained by the COMT genotype. In contrast, Haraldsson et al. (2010) failed to find a significant association in a similar study. However, they measured pursuit on continuous waveform motion without target blanking, so extraretinal processes might have been insufficiently prominent. Both studies provided only limited insights into differential links between the COMT Val^158^Met polymorphism and specific pursuit processes, because only a single paradigm was applied.

Our study provides insights into the effects of interindividual differences in dopaminergic transmission that do not go beyond the normal activity range, and moreover, affect individuals permanently across their lives. Therefore, comparison of our findings with results derived from pharmacological challenge studies in healthy subjects or clinical studies in schizophrenia patients requires some caution. However, we propose that, altogether, findings from different approaches can be reconciled.

There have been few studies concerned with effects of dopaminergic drugs on smooth pursuit eye movements in healthy subjects (Holzman et al., 1975; Malaspina et al., 1994; King et al., 1995). All studies exclusively considered steady-state pursuit on a continuously visible target, so retinal and extraretinal processes were not differentiated specifically. They provided inconsistent evidence for increased saccadic intrusions when dopaminergic activity is reduced. In particular, Malaspina et al. (1994) reported disrupted pursuit after administration of a potent dopamine antagonist, haloperidol. We suggest that these deficits are not inconsistent with our conclusion that dopamine modulates extraretinal, but not retinal, pursuit processes. Pursuit on a visible target relies on both low-level and high-level signals. If high-level contributions are massively blocked, disruptions, i.e., saccadic intrusions, can be expected. In contrast, more moderate modulations, as by dopaminergic polymorphisms, might not result in disrupted pursuit. Consistent with this speculation, studies that did not find increased saccadic intrusions after drug administration used much less potent dopamine antagonists than Malaspina et al. (1994) (Holzman et al., 1975; King et al., 1995).

Our findings are also congruent with clinical evidence from schizophrenia patients and the suggestion that pursuit deficits qualify for an endophenotype of the disease (Klein and Ettinger, 2008). Pursuit deficits were first documented more than 100 years ago (Diefendorf and Dodge, 1908), and since then an abundance of studies has accumulated confirming a pronounced dysfunction (Trillenberg et al., 2004; O’Driscoll and Callahan, 2008). Given massive dopaminergic dysregulation in schizophrenia (Abi-Dargham and Moore, 2003), it seems plausible to assume that dopamine contributes to these deficits; however, this association has remained ill defined because of the complexity of disease and confounding issues in the clinical setting. It is thus still controversial which specific processes contribute to pursuit deficits, but converging evidence strongly suggests that primarily extraretinal mechanisms are disturbed, whereas sensory driven processes are preserved (Thaker et al., 1996, 1998,1999; Avila et al., 2002; O’Driscoll and Callahan, 2008). It has been consistently shown that the typical findings of reduced gain and increased saccadic intrusions can be observed most reliably in paradigms emphasizing extraretinal pursuit processes. Whether the pursuit deficit in schizophrenia patients is modulated by the COMT polymorphism is still to be clarified (Rybakowski et al., 2002; Thaker et al., 2004; Haraldsson et al., 2009; Demily et al., 2016). Findings are heterogeneous and suggest that the COMT polymorphism plays a minor role when dopaminergic transmission is profoundly disturbed.

Because of the dominance of female subjects in our sample, the specific effects of the COMT genotype on high-level pursuit processes might represent a lower bound for modulation. Functional associations of the COMT Val^158^Met polymorphism are subject to sexual dimorphism (Tunbridge and Harrison, 2011; compare also Rybakowski et al., 2002). Most likely based on estrogenic regulation of COMT, the impact of the polymorphism has been demonstrated to be more consistent for men than for women (Chen et al., 2004; Barnett et al., 2007; White et al., 2014). Although our data do not allow any conclusions on sex-specific effects, we therefore suppose that more pronounced pursuit modulation could be expected in a sample with a balanced sex ratio. In summary, the significant association between the COMT Val^158^Met polymorphism and anticipatory pursuit processes in healthy subjects is not only consistent with the established functional model, but also provides a specific link between dopaminergic activity and high-level pursuit mechanisms.

The functional model of the SCL6A3 3′-UTR-VNTR polymorphism is also well established, and a critical effect on dopaminergic transmission in striatal areas is assumed (Costa et al., 2011; Faraone et al., 2014). Behavioral evidence, though, appears rather heterogeneous. Indeed, the polymorphism is associated with the risk for developing attention deficit hyperactivity disorder (Gizer et al., 2009), and many studies have investigated its effects on cognition and brain function in this patient group. However, meta-analytic studies indicate equivocal results (Rommelse et al., 2008; Dresler et al., 2014). Similarly, a recent meta-analysis of the association with cognition in healthy subjects could not find significant evidence (Ettinger et al., 2016). Given the pronounced and robust deficits in schizophrenia patients, we assumed smooth pursuit eye movements to be especially sensitive to dopaminergic modulation. In addition, striatal activity has been shown to contribute to pursuit control in monkeys (Basso et al., 2005).

Our results yet provide no support for a functional association between the SCL6A3 3′-UTR-VNTR polymorphism and any pursuit process. This finding qualifies the only previous report on a possible association by Wonodi et al. (2009). In the context of a clinical study on schizophrenia patients, they found a significant link between the polymorphism and predictive pursuit in their healthy control group. However, comparison of results is hampered by the specific genetic subgroups considered in the previous study. Results were based on the contrast between 10-repeat homozygous individuals, equivalent to the DAT9– subgroup in our study, and a group of individuals with diverse genotypes, including ∼20% rare genotypes that were not present at all in our sample. Thus, sample characteristics might have contributed to conflicting results.

We cannot exclude that in our data the absence of pursuit modulation by the SCL6A3 3′-UTR-VNTR polymorphism is based on a rather weak association not detectable with our given sample size. Moreover, it is also possible that oculomotor parameters after all are not sensitive enough to indicate rather small functional effects of some genetic polymorphisms. In several studies, for instance, genotype effects were not observable in behavioral measures, such as in the antisaccade task or stop-signal tasks, but clearly in brain function measured by BOLD activity (Ettinger et al., 2008; Congdon et al., 2009; Kasparbauer et al., 2015). Keeping these possibilities in mind, we conclude that our data do not provide any evidence of an association between the SCL6A3 3′-UTR-VNTR polymorphism and oculomotor control.

Notwithstanding the potential role of striatal dopamine activity in oculomotor control (compare Hikosaka et al., 2000 and Basso et al., 2005), our results positively pinpoint the dominant impact of prefrontal dopamine. Anticipatory pursuit in healthy observers is modulated by COMT activity in prefrontal cortex, but not by DAT availability in striatal areas. This functional localization agrees with the neuronal bases of anticipatory pursuit determined by single-unit recordings in monkeys (Hemptinne et al., 2008). Cortical dopamine activity, however, is embedded in a frontostriatal circuit involving downstream projections, and an inverse relationship between prefrontal and striatal dopamine activity has been suggested (Tunbridge et al., 2012). Although detailed dynamics of dopaminergic regulation elude direct investigation in noninvasive studies in humans, we tentatively assume that higher prefrontal dopamine activity triggers attenuated baseline striatal dopamine levels and thereby enhances efficiency of phasic striatal dopamine responses encoding anticipated rewards (Bayer and Glimcher, 2005; Gan et al., 2010). Two studies in rodents have only recently specified the functional properties of striatal dopamine (Hamid et al., 2016; Syed et al., 2016), emphasizing combined conveyance of action initiation, motivation, and learning. A boosted anticipatory pursuit response conforms perfectly to an assumed strengthening of these signaling characteristics. Taking the functional role of striatal transmission for granted, indirect downstream effects of prefrontal dopamine activity seem to be more powerful than individual differences because of the SCL6A3 3′-UTR-VNTR polymorphism that directly modulates striatal dopamine activity via DAT availability. It is understood that such a weighting has to be qualified by investigation of possible functional interactions of both polymorphisms. However, neither the present study nor previous studies have provided support for significant interactions (Caldú et al., 2007; Kasparbauer et al., 2015). To summarize, specific and localized functional effects of dopamine call attention to the fact that it is certainly not feasible to define a global role of dopamine for oculomotor control. Functionality is rather bound to specific dopaminergic subsystems and their embedding in the complex network of dopaminergic pathways in the brain.

Our approach and our results emphasize the value of systematic variability and individual differences for uncovering functional processes that would otherwise elude investigation (Vogel and Awh, 2008; Wilmer, 2008). Oculomotor control has been studied in extraordinary detail; nevertheless, there is still a striking discrepancy between the well-documented oculomotor deficits in diseases primarily characterized by disturbed neurotransmission and the scarce understanding of which mechanisms drive these deficits. For example, oculomotor deficits qualify as one of the most robust endophenotypes (vulnerability markers) of schizophrenia, but tentative genetic links are still to be discovered (Gottesman and Gould, 2003). Our data are congruent with a suggested oculomotor phenotype of the disease and furthermore provide insights into possible underlying mechanisms. Exploiting the effects of dopaminergic polymorphisms in healthy subjects allowed us to isolate circumscribed modulation of different pursuit processes and highlighted the particular impact of prefrontal dopamine on anticipatory mechanisms involved in pursuit. Previous studies identified dopamine receptors in frontal cortex as central for top-down control of early visual areas in monkeys (Noudoost and Moore, 2011). We provide a bridge between these findings and oculomotor deficits in patients with disturbed dopaminergic transmission. In healthy subjects, prefrontal dopamine activity plays a key role in the sophisticated interplay between bottom-up and top-down processes involved in smooth pursuit eye movements. These findings crucially contribute to a better understanding of how oculomotor control is modulated in healthy subjects.

## Synthesis

Reviewing Editor: Li Li, The University of Hong Kong Decisions are customarily a result of the Reviewing Editor and the peer reviewers coming together and discussing their recommendations until a consensus is reached. When revisions are invited, a fact-based synthesis statement explaining their decision and outlining what is needed to prepare a revision will be listed below. The following reviewer(s) agreed to reveal their identity: Stephen Heinen, Drion Liston.

The ms is potentially interesting for publication. Reviewer 1 has a serious concern regarding the interpretation of the data that should be successfully addressed in the revision before the ms can be considered for publication. I enclose Reviewer 1's comments as given below:

While most of the results are straightforward and the conclusions are potentially interesting, there is a critical flaw in the interpretation of the data. Specifically, the conclusion that dopamine does not influence visual pursuit appears to be contradicted by the data they present in Figure 3. Visual pursuit onset latency (3F) and the parameters of catch-up saccades (3G, 3H), which are also guided by the visual target, show a clear modulation with phenotype.

The caption for Figure 3C alludes to temporal variability between observers, but it would be helpful to be more specific. I assume there was more temporal variability in the Met/Met group than the Val/Met group, and hence the averaged velocity peak of the former appeared lower than that of the latter in figure 3B because the peak was smeared, and hence its amplitude reduced by the averaging. Perhaps the authors could add a sentence to that effect in the results. In addition, "amplitude lag" in Figure 3H is not standard terminology; do the authors mean "error"?

Reviewer 2 also has some concerns that should be addressed in addition to some constructive suggestions to improve the clarity as well as the quality of the manuscript. I enclose Reviewer 2's comments as given below:

Major points.

Let me first say that I liked this paper; it should be published. I liked the approach, the methods, and the data. Some of the labels and framing might need some more thought, however, and it seemed like the data picture was incomplete.

1. Some of the language associated with the framing of the experimental design and interpretation of the results seem stretched. As a result, some of conclusions, even at the level of the abstract, seemed tenuous. For starters on this point, I would ask the authors to consider why they used the term "anticipatory", rather than "expected", or to take this argument one step further, "perceived". Consider two possible perceptual interpretations of the "implied motion" task: first, the black target spot disappears at a fixed velocity behind an occluder then reappears in the periphery; second, the spot remains at the original fixed position but disappears entirely, only to appear some time later moving in the periphery. Clearly, based on the context suggested by the other trials, the first interpretation is more likely (Law of Pragnanz) and seems consistent with the observed oculomotor patterns, as the second interpretation wouldn't be expected to drive eye movements.

At times, the authors use a generic term "top-down" or "high-level" to refer to this type of signal - either of which may be more accurate. Bob Wurtz coined the term "extraretinal", which may also work. The major high-vs-low distinction the I think the authors are (or perhaps should be) alluding to goes back to Robinson (Robinson, Gordon & Gordon, 1986) and Larry Young (Yasui and Young, 1975): the transition from "retinal-slip-driven pursuit initiation" and steady-state pursuit driven by some "higher-level" signal, possibly related to perceived object motion. The "implied motion" task assesses the ability of a similar (possibly this same) "high-level" signal to initiate and sustain eye velocity in the absence of visual motion, which changes markedly across Val/Met cohorts. From there, one might suggest that dopaminergic pathways may contribute to, support, or carry this high-level signal guiding smooth pursuit to implied target motion. Interesting, but the readers will want to see whether there are ANY EFFECTS AT ALL on any known metrics of more traditional visual tracking (see point 3).

2. For better or worse, if schizophrenia is described as a motivation for this experiment, the manuscript will need to discuss these results in the context of schizophrenia in some depth, even if the answers aren't yet clear-cut. Here are some questions that come to mind: Is the Val/Met polymorphism a risk factor for schizophrenia? Are Val/Met polymorphisms associated linked to any psychiatric illness? What is known about other differences between Val/Met polymorphism cohorts? Personality traits, anything? How do the classic smooth pursuit effects of schizophrenia, low-gain pursuit and large, frequent catch-up saccades relate to dopaminergic dysfunction? The main conclusion from this paper is that prefronal dopamine (Fig. 1A) improves all measured tracking metrics to implied motion (Fig. 3). Consistent with this, Malaspina et al. paper haloperidol to induce saccadic pursuit deficits in normals, but to visible motion. Yet, we see no effect of the Val/Met polymorphism on tracking of visible motion (Fig. 5). Discuss.

3. The readers will expect to see the EXACT SAME ANALYSIS for the negative controls and the two experimental conditions. Please replicate Fig. 3 for the DAT9+/-. Consider adding the two new green bars to each set of axes in Fig 3. If we think of DAT+/- as a nominal "control" factor, it would be interesting to see those bars plotted side-by-side at the same level as the heterozygote cohort.

Also, please replicate the Fig. 3 analysis for the visually-guided task, for the Val/Met cohort and for the DAT9 cohorts? Consider putting them all in one figure like Fig. 3, with three blue bars and two green bars per set of axes. Panels A and B can be split vertically to accommodate VAL/Met and DAT9, or plot them all together. We need a more complete picture of this. Also, please consider adding gain (as per Fig. 5) to Fig. 3. One other metric of interest is proportion smooth, the ratio of smooth pursuit eye displacement to total (saccadic plus pursuit) eye displacement during a defined steady-state tracking interval (Liston & Stone, 2014). Overall, I want to be reassured that, as you say, the Val/Met polymorphism shows no effect on any performance metric for visual tracking.

4. Just to get some sense for the signal-to-noise ratio of Val/Met effect, please consider adding a table for the pairwise ROC areas across Val/Mat cohorts, for all metrics.

Minor points.

Fig. 2. Is this really necessary?

Line 34: "Pursuit paradigms were chosen to assess the ability of the pursuit system to sustain tracking during target blanking,"

Line 50: "to investigate the effect of individual differences in dopaminergic circuitry on smooth pursuit eye movements"

Lines 62-63: "is considered to be the major pathologic mechanism" "clinical findings are mixed and links to dopamine are tenuous."

Line 67: "By pharmacological manipulation of prefrontal dopaminergic transmission it was possible to modulate visual cortical signals in area V4 that contribute to saccadic target selection."

Line 206: Why didn't you use a step-ramp, rather than a ramp?

Why were there 106, rather than 110, SCL6CA subjects? Did I miss something in the methods?

Fig. 2. Darken the 0-500 ms time band, it is barely perceptible.
